# The moderating role of food cravings in the relationship between weight suppression and eating disorder psychopathology in college students

**DOI:** 10.3389/fpsyg.2024.1343048

**Published:** 2024-09-06

**Authors:** Susana Cruz Garcia, Julia M. Hormes

**Affiliations:** Department of Psychology, University at Albany, State University of New York, Albany, NY, United States

**Keywords:** food cravings, weight suppression, binge eating, purging, college students

## Abstract

Weight suppression (WS), the difference between an individual’s current and highest adult weight, is predictive of eating-related pathology across diagnostic categories and poor eating disorder treatment outcomes, but findings from non-clinical samples have been mixed. Cravings are strong urges for specific foods that are subjectively difficult to resist. Food cravings are now widely conceptualized as cognitive-affective states characterized by intrusive thoughts that are perceived as distressing and can interfere with adaptive functioning. Food cravings are known antecedents of binge eating, but little is known about how they interact with WS. We examined the obsessive-compulsive aspects of food cravings as potential moderators of the association between WS and eating disorder symptoms in general, and binge eating specifically in a cross-sectional study of college students. Participants (*n* = 144, 60.4% female) self-reported their height and current and past highest adult weight and completed the Binge Eating Scale (BES), Eating Disorder Examination-Questionnaire (EDE-Q), and Obsessive Compulsive Eating Scale (OCES). Main and interactive effects of WS and OCES scores on EDE-Q global and BES scores were examined in regression models. There were significant main effects of OCES scores on overall eating disorder symptom severity as well as binge frequency, with those endorsing more craving-related intrusive thoughts and compulsive urges engaging in maladaptive behaviors more frequently. WS alone did not consistently cross-sectionally predict eating disorder psychopathology. Findings suggest that food cravings are significantly associated with disordered eating symptoms and specifically binge eating frequency and should be accounted for in future research on WS in clinical and non-clinical samples.

## Introduction

Binge eating is prevalent in clinical and non-clinical samples and associated with significant psychological and physical sequelae (Hudson et al., 2007; Marques et al., 2011; Dingemans and van Furth, 2012; Stice et al., 2013; Mustelin et al., 2015; Duncan et al., 2017; Thornton et al., 2017; Hutson et al., 2018; Guerdjikova et al., 2019; Keski-Rahkonen, 2021; Qian et al., 2022). Weight suppression (WS), the difference between one’s highest and current adult weight outside of pregnancy, is a robust predictor of binge and loss of control eating in clinical samples and of poor bulimia nervosa treatment outcomes (Butryn et al., 2006, 2011; Dawkins et al., 2013; Bodell and Keel, 2015; Accurso et al., 2016). Notwithstanding, findings regarding links between WS and binge and loss of control eating in non-clinical samples remain inconsistent. For example, WS predicted the onset and maintenance of binge eating at a ten-year follow-up in one study of college students (Keel and Heatherton, 2010). On the other hand, three community-based studies found WS to be unrelated to current binge eating or the odds of future binge eating disorder onset (Van Son et al., 2013; Stice et al., 2020; Forney et al., 2023).

Identification of moderators of the relationship between WS and binge eating may help clarify inconsistent prior findings. Lowe et al. (2007) examined the moderating role of dietary restraint and found a direct effect of WS on binge eating, but an inverse relationship between dietary restraint and binge eating frequency, with no significant interaction (Lowe et al., 2007). Butryn et al. (2011) established body mass index (BMI) as a significant moderator of the relationship between WS and binge eating, with participants with low BMI and high levels of WS binge eating most frequently (Butryn et al., 2011). Both studies examined WS and binge eating cross-sectionally in clinical samples of women with bulimia nervosa. Burnette et al. (2017) examined gender as a moderator of the relationship between WS and disordered eating behaviors cross-sectionally in undergraduate students and found WS to be indirectly related to loss of control eating through dietary restraint in both men and women, while high levels of WS were associated with greater frequency of vomiting and laxative use specifically in men (Burnette et al., 2017). A recent study of three non-clinical samples did not find WS to be significantly associated with binge eating frequency, but identified weight history (i.e., lifetime highest and lowest weight) as a possible moderator of this relationship such that a history of higher weight appeared to buffer against the adverse effects of WS on binge eating frequency (Forney et al., 2023). More work is needed to identify additional moderators of the impact of WS on disordered eating behaviors in non-clinical populations.

An unexplored variable that may potentially moderate the link between WS and binge eating and help understand inconsistencies in prior research is food cravings. Food cravings are strong urges to consume specific foods that are subjectively difficult to resist (Rogers and Smit, 2000; Hormes and Rozin, 2010). There is substantial evidence of food cravings as significant predictors of binge eating in clinical and non-clinical samples (Greeno et al., 2000; Waters et al., 2001; Jarosz et al., 2007; Ng and Davis, 2013; Innamorati et al., 2014; Leslie et al., 2018; Meule et al., 2018). Indeed, food cravings appear to be a defining feature that differentiates individuals with and without binge eating disorder (Ng and Davis, 2013). Food cravings are now widely conceptualized as cognitive-affective states. For example, the Elaborated Intrusion theory of desire (“EI Theory”) views cravings as the result of a two-step process: first, automatic thoughts about the target food appear, prompted by external or internal cues, and second, these intrusive thoughts are actively elaborated upon, a process that maintains the craving and often results in consumption of the desired food (May et al., 2004; Kavanagh et al., 2005; May et al., 2015; Schumacher et al., 2017). It is thought that avoidance of highly palatable foods or even avoidance of thoughts about these foods may paradoxically strengthen the intensity of cravings by facilitating elaboration and increasing the salience of craving-related cognitions (Erskine and Georgiou, 2010; Hooper et al., 2012; Massey and Hill, 2012).

There is a theoretical rationale as well as preliminary empirical evidence to support a role of food cravings in the relationship between WS and binge eating. Weight suppression is thought to increase risk of bulimic-type symptoms in part because of the conscious effort required to engage in the dieting and compensatory behaviors needed to maintain a suppressed weight (Butryn et al., 2011). Similarly, food cravings are thought to be exacerbated by weight loss dieting as well as active attempts to avoid or restrict highly palatable yet seemingly forbidden or avoided foods (Cartwright and Stritzke, 2008; Hormes and Timko, 2011; Massey and Hill, 2012; Meule, 2020). In other words, highly weight suppressed individuals are likely to experience elevated levels of food cravings, which may contribute to increased risk of binge eating. Indeed, a recent study found that highly weight suppressed individuals endorse elevated levels of approach-avoidance of high-calorie food compared to a low weight suppressed group, which is a known mechanism underlying strong urges for those foods (Lee and Lee, 2021). Specifically, in the context of EI Theory, conscious efforts to avoid or suppress thoughts related to well-liked or craved targets paradoxically increase their salience, facilitate elaboration, and contribute to the maintenance and strengthening of the craving episode and greater likelihood of subsequent binge eating episodes (Barnes and Tantleff-Dunn, 2010).

### Study aims

Despite the established role of food cravings as a predictor of consumption, evidence to link WS to binge eating, and potential similarities in the hypothesized mechanisms underlying these constructs, research has not explored whether WS and food cravings interact to impact binge eating. The primary aim of this cross-sectional study was to test the hypothesis that food cravings moderate the relationship between WS and binge eating in a non-clinical sample of undergraduate students, who are at elevated risk for the onset and exacerbation of disordered eating behaviors, especially since the COVID-19 pandemic (Harrer et al., 2020; Tavolacci et al., 2021). Identification of moderators of the relationship between WS and binge eating can inform prevention and treatment interventions to reduce the frequency and severity of binge eating.

While the main focus of work on WS has been on its role as a predictor of binge and loss of control eating, preliminary evidence suggests that it may be more broadly predictive of disordered eating behaviors and cognitions across diagnostic categories, including weight and shape concerns, excessive exercise, restrictive behaviors, and use of weight control medications (Lavender et al., 2015). As a secondary aim, we therefore also explored main and interactive effects of WS and craving on general eating disorder pathology as quantified via the Eating Disorder Examination-Questionnaire.

## Materials and methods

Methods were reviewed and approved by the local Institutional Review Board (protocol #17E119). Participants reviewed an informed consent form describing the purpose and voluntary and anonymous nature of the research prior to the completion of questionnaires. To protect participant confidentiality, a waiver of signed informed consent was obtained; participants indicated their consent to participate by advancing to the next page of the survey after reviewing the informed consent form.

### Participants

Participants (*n* = 144, 60.4% female, *M* = 18.87 years old, *SD* = 1.09, range: 18–24) were undergraduate students recruited from the research participant pool of a large public university in the northeastern United States. This sample size is comparable to or exceeds the number of participants included in similar prior investigations of moderators of the relationship between WS and binge eating (Lowe et al., 2007; Butryn et al., 2011). Participants self-identified (in overlapping percentages) as White (48.6%, *n* = 70), Black/African American (29.2%, *n* = 42), Hispanic or Latino/Latina (16.7%, *n* = 24), Asian (13.9%, *n* = 20), American Indian or Alaskan Native (1.4%, *n* = 2), and “other/unspecified” race or ethnicity (1.4%, *n* = 2).

### Procedures

Data were collected as part of a larger study of eating and health behaviors. Measures analyzed here were included for the specific purpose of examining the relationships between WS, food cravings, and eating disorder psychopathology. All measures were completed online via the secure server Qualtrics. Participants received course credit upon completion of the study.

### Measures

#### Traditional, developmental, and percent weight suppression

Participants self-reported current height and current and highest weight at current height (outside of pregnancy or illness) to calculate indices of WS. Consistent with prior work, we measured WS conventionally as the difference between participants’ self-reported highest past minus current weight at adult height (Lowe et al., 2007; Bodell and Keel, 2015; Lee and Lee, 2021). To address concerns about possible limitations of this traditional index of WS, especially when it comes to exploring relationships between WS and eating disorder pathology (Schaumberg et al., 2016), we also calculated two additional indices of weight suppression. Developmental weight suppression (DWS) reflects the difference between participants’ highest and current z-body mass index (BMI), taking into account developmentally-relevant factors of sex, age, and height (Singh et al., 2021). “Percent weight suppression” (% WS) was calculated as the difference between highest lifetime and current weight divided by highest weight, an approach that has been shown to reduce skew and the influence of highest lifetime weight (Schaumberg et al., 2016; Forney et al., 2023). Analyses reported here were conducted with all three indices of WS (in separate regression analyses); results for the DWS and % WS measures are only reported if they deviated from findings derived using the traditional index of WS.

#### Obsessive compulsive eating scale (OCES)

Based on evidence for phenomenological and neuroanatomical overlaps between cravings for various substances and obsessive-compulsive disorder (Anton, 2000), the Obsessive Compulsive Eating Scale seeks to capture the obsessive-compulsive aspects of food cravings, quantifying obsessive and repetitive thoughts and excessive preoccupations with the craving target along with impulsive urges and subjective lack of control over the problem behavior (Niemiec et al., 2016). The measure is part of a larger family of craving measures derived from the well-validated and widely used Yale-Brown Obsessive Compulsive Scale and may be especially suitable for quantifying the adverse impact of craving-related intrusive thoughts on adaptive functioning, compared to more traditional craving measures that are primarily focused on assessing the frequency, intensity, and specific targets of cravings (Goodman et al., 1989; Anton, 2002; Franken et al., 2002; Hormes et al., 2012; Niemiec et al., 2016). The focus of the OCES on capturing intrusive thoughts associated with craving states is theoretically consistent with EI Theory, which provides the theoretical framework that guides the present work.

Respondents are instructed to complete the 14-item scale while thinking about a frequently craved food that they are actively trying to avoid consuming. As such, the OCES quantifies the cognitive underpinnings of food cravings in a manner that is theoretically consistent with EI Theory in terms of intrusive thoughts and compulsive urges that are thought to be made more salient by active efforts to avoid the craving target. Items are rated on a scale of 0 to 4 and quantify the “obsessive” (e.g., “How successful are you in stopping or diverting these thoughts when you are avoiding this food?”) and “compulsive” (e.g., “How much does avoiding this food interfere with your social functioning? Is there anything that you do not or cannot do because of your avoiding this food?”) tendencies of thoughts and urges underlying specific food cravings. Higher scores indicate increased distress and interference of thoughts and urges related to cravings. Internal consistency reliability was good or excellent for the “obsessive” (Cronbach’s α = 0.86) and “compulsive” (0.86) subscale and total OCES scores (0.93).

In prior work, the OCES demonstrated good convergent validity with established measures of general and specific food cravings (i.e., the Food Craving Questionnaire – Trait – reduced and the Food Craving Inventory) (White et al., 2002; Hormes and Meule, 2016), as well as criterion validity, successfully differentiating between binge eaters and non-binge eaters as identified using the Binge Eating Scale (Niemiec et al., 2016). In the present study, OCES total (*r* = −0.34, *p* < 0.001) and “obsessive” (*r* = −0.36, *p* < 0.001) and “compulsive” subscale scores (*r* = −0.31, *p* < 0.001) were significantly and inversely correlated with scores on the Food Craving Acceptance and Action Questionnaire, another measure of the cognitive underpinnings of food cravings that quantifies acceptance of urges to consume craved foods (Juarascio et al., 2011).

#### Binge eating scale (BES) (Gormally et al., 1982)

The 16-item BES was used to assess the behavioral, affective, and cognitive manifestations associated with binge eating (Cronbach’s *α* = 0.91). Higher total scores indicate greater eating pathology. The BES was used here because it has been shown to be especially suitable for capturing clinically significant binge eating in non-clinical samples (Duarte et al., 2015; Brunault et al., 2016).

#### Eating disorder examination questionnaire (EDE-Q) (Fairburn and Cooper, 1993)

General eating disorder symptom severity was quantified via the EDE-Q, a widely used, well-validated, and comprehensive assessment of eating disorder pathology suitable for use with clinical samples as well as the general population (Mond et al., 2004). The EDE-Q global score is calculated as the average of the restraint, eating, shape, and weight concern subscales. Internal consistency reliability in the present sample was excellent (Cronbach’s α = 0.93).

### Statistical analyses

Analyses were performed using IBM SPSS v.27. Participants under the age of 18 (*n* = 1) or not indicating their age (*n* = 2) were excluded from the analyses. Instructions for completion of the OCES indicate that only participants currently making any effort to restrict consumption of a specific food or food group should provide responses (with the assumption that such avoidance paradoxically increases the frequency and intensity of craving-related thoughts). Consistent with these guidelines, only participants meeting criteria for completion of the OCES by endorsing current attempt to avoid or limit consumption of craved foods (*n* = 144, 36.4% of the entire sample) were included in the analyses reported here.

We conducted moderated regressions analyses to examine if food cravings, WS, or their interaction predicted BES or EDE-Q global scores. Continuous variables were mean centered before inclusion in the regression models. Gender was entered as a covariate in Step 1, followed by main effects in Step 2, and the interaction term in Step 3. Missing data for WS was 1.4, 15.3% for the OCES (ranging from 0.7% on item 1 to 10.4% on item 8), 13.9% for the BES (ranging from 0.7% on items 1, 2, 3, and 5 to 4.2% on item 8), and 15.3% for the EDE-Q global score (ranging from 0.7% for items 1 and 10 to 4.2% for item 8). Skewness was <2 for indices of WS and OCES, BES, and EDE-Q scores.

## Results

Mean WS in the present sample was 8.31 lbs. (*SD* = 8.89). The three indices of WS were significantly and positively correlated with one another (see Table 1). The traditional index of weight suppression was also significantly and positively correlated with current and highest BMI. There were no significant gender differences in indices of WS, current or highest BMI, or OCES scores; women reported more symptoms of disordered eating than men as reflected in significantly higher EDE-Q global scores as well as significantly higher scores on the BES (see Table 1). OCES total scores were significantly and positively correlated with EDE-Q global and BES total scores (see Table 1).

**Table 1:** Descriptives, comparisons by gender, and Pearson’s product moment correlations between key variables.

		Female (*n* = 86) *M* (*SD;* range)	Male (*n* = 56) *M* (*SD*)	Statistic	WS (lbs)	DWS	Percent WS	OCES total score	BES	EDE-Q global score
WS (lbs)		7.53 (6.79; 0–30)	9.52 (11.36; 0–50)	*t*(140) = 1.18, *p* = 0.24, *d* = 0.21	*–*	*r* = 0.80, *p* < 0.001	*r* = 0.95, *p* < 0.001	*r* = −0.06, *p* = 0.53	*r* = 0.14, *p* = 0.11	*r* = 0.19, *p* = 0.12
DWS		0.24 (0.21; 0–0.91)	0.28 (0.30; 0–1.17)	*t*(135) = 0.81, *p* = 0.42, *d* = 0.15			*r* = 0.90, *p* < 0.001	*r* = −0.09, *p* = 0.36	*r* = 0.09, *p* = 0.33	*r* = −0.05, *p* = 0.71
% WS		0.05 (0.04)	0.05 (0.05)	*t*(140) = 0.17, *p* = 0.86, *d* = 0.00				*r* = −0.05, *p* = 0.57	*r* = 0.18, *p* = 0.05	*r* = 0.15, *p* = 0.22
OCES	Total score	0.67 (0.62; 0–2.21)	0.57 (0.68; 0–2.64)	*t*(120) = 0.83, *p* = 0.40, *d* = 0.15					*r* = 0.51, *p* < 0.001	*r* = 0.28, *p* =0.03
	“Obsessive”	0.67 (0.68; 0–2.67)	0.56 (0.71; 0–2.67)							
	“Compulsive”	0.67 (0.62; 0–2.25)	0.57 (0.70; 0–2.75)							
BES		12.99 (9.48; 0–40)	9.54 (6.92; 1–24)	*t*(122) = 2.34, *p* = 0.02, *d* = 0.42						*r* = 0.68, *p < 0.*001
EDE-Q	Global score	1.70 (1.14; 0–4.23)	1.23 (0.85; 0.0–3.43)	*t*(120) = 2.40, *p* = 0.01, *d* = 0.47						
Current BMI (kg/m^2^)		24.47 (5.58; 16.25–50.66)	24.53 (3.58; 17.54–34.99)	*t*(139) = 0.08, *p* = 0.94, *d* = 0.01	*r* = 0.24, *p = 0.*01	*r* = −0.22 *p = 0.*01	*r* = 0.06, *p* = 0.44	*r* = 0.04, *p = 0*.66	*r* = 0.11, *p* = 0.25	*r* = 0.36, *p* = 0.002
Highest BMI (kg/m^2^)		25.78 (6.05; 16.25–54.32)	25.85 (4.31; 17.54–37.59)	*t*(137) = 0.08, *p* = 0.94, *d* = 0.01	*r* = 0.46, *p < 0.*001	*r* = 0.01, *p = 0.*88	*r* = 0.30, *p* < 0.001	*r* = 0.02, *p = 0*.76	*r* = 0.15, *p* = 0.11	*r* = 0.39, *p* = 0.001

DWS, developmental weight suppression; WS, weight suppression; % WS, percent WS/WS divided by highest lifetime weight; BMI, body mass index; OCES, Obsessive Compulsive Eating Scale; BES, Binge Eating Scale; EDE-Q, Eating Disorder Examination – Questionnaire.

Regression analysis revealed significant main effects for WS and OCES craving scores on binge eating frequency as quantified via the BES, but no interaction (see Table 2). The main effect of WS on BES scores was no longer significant when using the DWS index as a predictor variable. Percent WS and OCES total scores were both significant cross-sectional predictors of BES total scores, with no interaction (see Table 2).

**Table 2:** Moderated regression analyses assessing main and interactive effects of food cravings and weight suppression on binge eating scale and EDE-Q global scores.

	Binge eating scale						
	*Β* (95% CI)	*β*	*t*	*R* ^2^	Δ*R*^2^	ΔF	df
Gender	−3.90 (−6.76, −1.03)	−0.22	−2.38*	0.05	0.05	5.68*	105
WS	0.19 (0.04, 0.34)	0.20	2.46*	0.34	0.28	21.95*	103
OCES Total	6.96 (4.71, 9.21)	0.50	6.14*				
WS × OCES	−0.16 (−0.43, 0.12)	−0.09	−1.15	0.34	0.01	1.33	102

	Binge eating scale						
	*B* (95% CI)	*β*	*t*	*R* ^2^	Δ*R*^2^	ΔF	df
Gender	−3.32 (−6.14, −0.49)	−0.19	−2.32*	0.05	0.05	5.68*	105
% WS	38.18 (7.08, 69.27)	0.20	2.44*	0.33	0.28	21.86***	103
OCES Total	6.67 (4.58, 9.16)	0.49	5.95***				
WS × OCES	−25.55 (−76.22, 25.11)	−0.08	−1.00	0.34	0.01	1.00	102

	EDE-Q global score						
	*B* (95% CI)	*β*	*t*	*R* ^2^	Δ*R*^2^	ΔF	df
Gender	−0.47 (−0.86, −0.07)	−0.21	−2.35*	0.05	0.05	4.89*	0.102
WS	0.02 (0.003, 0.05)	0.20	2.24*	0.21	0.17	10.69*	100
OCES total	0.67 (0.33, 1.01)	0.35	3.91*				
WS × OCES	−0.009 (−0.05, 0.03)	−0.04	−0.46	0.22	0.002	0.21	99

OCES, obsessive compulsive eating scores; WS, weight suppression; % WS, percent WS/WS divided by highest lifetime weight; B, β and t reflect values from the final regression equation; *p < 0.05.

Regression analysis similarly revealed significant main effects for WS and OCES craving scores on EDE-Q global scores, but no interaction (see Table 2). The main effect of WS on EDE-Q global scores was no longer significant when using the DWS or percent WS indices as predictor variables.

To address potential concerns about missingness, we reran analyses using imputed data (multiple imputation with five iterations for each imputation). In these revised analyses, the main effects of WS on BES and EDE-Q global scores and of percent WS on BES scores were no longer significant. Otherwise, all results from multiple regression analyses remained the same in terms of significant main and interaction effects.

## Discussion

This study aimed to add to our understanding of the impact of WS on disordered eating symptoms, and specifically binge eating frequency in a non-clinical sample of undergraduate students. Food cravings and WS have been linked to binge eating independently and may share underlying mechanisms (Lowe et al., 2007; Ng and Davis, 2013; Van Son et al., 2013; Chao et al., 2016; Leslie et al., 2018; Meule et al., 2018; Lee and Lee, 2021). Specifically, the active effort required to limit consumption and maintain a suppressed weight appears may involve cognitive processes similar to those now widely believed to be involved in the emergence and maintenance of food cravings, including avoidance and suppression of thoughts related to desirable foods, which paradoxically increase the salience of these thoughts and the likelihood of binge eating (Lee and Lee, 2021). We thus hypothesized that food cravings moderate the relationship between WS and binge eating frequency. As a secondary aim, we also sought to explore main and interactive effects of WS and craving on disordered eating symptoms more generally.

As hypothesized and consistent with prior work, indices of food cravings and WS were independently cross-sectionally predictive of binge eating. The main effects of food craving were consistent across all analyses conducted, with more mixed findings regarding the effects of WS. Specifically, while the traditional and percent indices of WS were significantly associated with binge eating frequency as quantified via the BES, this relationship was no longer significant when using developmental WS as the predictor variable. Weight suppression and food craving scores were also significantly associated with general eating pathology as quantified via the EDE-Q global score, with no evidence for an interaction effect. Once again, the main effect of OCES scores was robust, while the main effect of WS on EDE-Q global scores was no longer significant when using the alternative developmental or percent WS indices as predictor variables. Contrary to our hypothesis, there was no evidence for a role of food craving as a moderator of the relationship between WS and binge eating frequency or general eating disorder symptom severity. Taken together, these findings confirm the powerful impact of the intrusive thoughts and distress associated with food cravings on binge eating and overall eating disorder pathology. Results also highlight the importance of accounting for these cognitive underpinnings of food cravings in future studies of WS and eating disorder psychopathology and could help explain some of the inconsistent findings in past studies of the impact of WS on eating behaviors.

To our knowledge, this is the first study looking at food cravings in relation to WS and binge eating in a diverse population of undergraduate students, who are at elevated risk for the onset of disordered eating (Goldschmidt et al., 2011; Kessler et al., 2013). Strengths of the current study included our nuanced assessments of the constructs of interest, with selected measures representing current views of craving etiology, accounting for developmental factors and highest prior weight in our calculations of WS, and considering the potentially unique presentation of binge eating in non-clinical samples. Some limitations to the study must also be noted, including the cross-sectional nature of the study design. Constructs of interest were quantified using self-report, which may be subject to bias. Mean WS was lower than what is typically documented in eating disorders samples (i.e., over 15 lbs.) (Lowe et al., 2006; Carter et al., 2015; Lowe et al., 2018), but similar to other non-clinical college and community samples (i.e., under 10 lbs.) (Bodell et al., 2017; Burnette et al., 2017). The extent to which findings can be replicated in samples with more significant eating pathology should be explored in future research.

In conclusion, our data replicate strong associations between food cravings and binge eating frequency and general eating disorder pathology, with no evidence for a moderating role of food craving in the relationship between WS and binge eating. Findings have important clinical implications to help elucidate points for preventive intervention and specifically suggest that a better understanding of the cognitive underpinnings of food cravings and WS can improve current treatments of conditions characterized by binge eating, restraint, and weight, shape, and eating concerns. Mindfulness-based approaches have been shown to be effective in reducing the adverse impact of cravings on behaviors across a range of domains, including food, alcohol, and substance use (Witkiewitz et al., 2013; Brewer et al., 2014; Lacaille et al., 2014; Tapper, 2018). Future research should explore the utility of these approaches in reducing binge eating frequency and severity in highly weight suppressed individuals with comorbid food cravings.

## Data availability statement

The raw data supporting the conclusions of this article will be made available by the authors, without undue reservation.

## Ethics statement

The studies involving humans were approved by Institutional Review Board at the University at Albany. Participants reviewed an informed consent form describing the purpose and voluntary and anonymous nature of the research prior to the completion of questionnaires. To protect participant confidentiality, a waiver of signed informed consent was obtained; participants indicated their consent to participate by advancing to the next page of the survey after reviewing the informed consent form.

## Author contributions

SG: Conceptualization, Data curation, Formal analysis, Methodology, Visualization, Writing – original draft. JH: Conceptualization, Data curation, Formal analysis, Methodology, Project administration, Resources, Supervision, Visualization, Writing – review & editing.
